# Eco-bio-social research on dengue in Asia: a multicountry study on ecosystem and community-based approaches for the control of dengue vectors in urban and peri-urban Asia

**DOI:** 10.1179/2047773212Y.0000000055

**Published:** 2012-12

**Authors:** Johannes Sommerfeld, Axel Kroeger

**Affiliations:** 1 Programme for Research and Training in Tropcial Diseases (TDR), World Health Organization, Geneva, Switzerland; 2 Liverpool School of Tropical Medicine, Liverpool, UK

**Keywords:** Dengue, Vector-borne diseases, EcoHealth, Eco-bio-social research, Intersectoral, Community-based interventions, Urban and peri-urban Asia, India, Indonesia, Myanmar, Philippines, Sri Lanka, Thailand, Integrated vector management, IVM

## Abstract

This article provides an overview of methods and cross-site insights of a 5-year research and capacity building initiative conducted between 2006 and 2011 in six countries of South Asia (India, Sri Lanka) and South-East Asia (Indonesia, Myanmar, Philippines, Thailand).The initiative managed an interdisciplinary investigation of ecological, biological, and social (i.e., eco-bio-social) dimensions of dengue in urban and peri-urban areas, and developed community-based interventions aimed at reducing dengue vector breeding and viral transmission. The multicountry study comprised interdisciplinary research groups from six leading Asian research institutions. The groups conducted a detailed situation analysis to identify and characterize local eco-bio-social conditions, and formed a community-of-practice for EcoHealth research where group partners disseminated results and collaboratively developed site-specific intervention tools for vector-borne diseases. In sites where water containers produced more than 70% of *Aedes* pupae, interventions ranged from mechanical lid covers for containers to biological control. Where small discarded containers presented the main problem, groups experimented with solid waste management, composting and recycling schemes. Many intervention tools were locally produced and all tools were implemented through community partnership strategies. All sites developed socially and culturally appropriate health education materials. The study also mobilised and empowered women’s, students’ and community groups and at several sites organized new volunteer groups for environmental health. The initiative’s programmes showed significant impact on vector densities in some sites. Other sites showed varying effect — partially attributable to the ‘contamination’ of control groups — yet led to significant outcomes at the community level where local groups united around broad interests in environmental hygiene and sanitation. The programme’s findings are relevant for defining efficient, effective and ecologically sound vector control interventions based on local evidence and in accordance with WHO’s strategy for integrated vector management.

## Introduction

Dengue is the fastest advancing vector-borne arboviral disease in terms of geographical expansion and number of cases and is a significant economic and social burden in many countries worldwide.^1^,^2^ The breeding sites of dengue’s predominant vector, *Aedes aegypti* — water containers of different types present in domestic and peridomestic environments — are closely related to environmental factors linked to and maintained by human behavior. Recrudescent dengue is a particular public health concern in Asian cities and peri-urban areas where the disease is now widely endemic and where epidemic outbreaks occur with increasing frequency and intensity. Dengue emergence and resurgence is widely associated with rapid and uncontrolled urbanization.^3^,^4^ Complex transmission patterns in urban environments are particularly challenging for control efforts. Context-specific vector control efforts urgently need to advance intersectoral partnerships, engage local communities, and harmonize with principles of integrated vector management (IVM).

Dengue prevention relies on vector control and will likely continue to do so even when an effective dengue vaccine becomes available. In the past 10 years, significant progress was made in developing innovative biological, chemical, and mechanical vector control technologies.^5^ Recent research suggests that genetic modification of insect vectors is a potentially effective control method, and addresses ethical, legal and social implications of the technology’s field deployment.^6^,^7^

Vector control tools, regardless of their technological basis, must be feasible and practical to apply in real-life situations. Community engagement and intersectoral partnerships are particularly important elements of integrated public health strategies for vector control. In Asia, government services routinely conduct vector control using the characteristically ‘top-down approach’ of vertical programmes that consist of larviciding water containers and space spraying of insecticides in neighborhoods with reported dengue cases. Novel approaches to dengue vector management successfully tested in recent years include targeted interventions in key productive container types (as determined by pupal indices)^8^ in combination with insecticide treated window curtains and/or water container covers.^9^

Existing research on the complexity of eco-bio-social contexts repeatedly argues that dengue control necessitates sound intersectoral approaches that combine environmental management practices with community mobilization.^10^–^14^ Behaviour change interventions are considered central,^11^ but they have long focused on changing knowledge and beliefs, educating communities and enrolling them into community-based vector control efforts aimed at larval production site elimination and management.

The research presented in this special issue results from a multicountry research initiative in Asia, carried out between 2006 and 2011, supported by a research and capacity building partnership between the Special Programme for Research and Training in Tropical Diseases (TDR) and the Ecosystem and Human Health Program of Canada’s International Development Research Centre (IDRC).The overall objective of the research programme was to strategize and contribute to improved dengue prevention using interdisciplinary analysis to better understand ecosystem-related, biological, and social determinants of dengue, as well as to develop and evaluate intersectoral and community-centered ecosystem management interventions directed at reducing dengue vector habitats. This multicountry research initiative complements earlier research programmes facilitated by TDR that substantiated the cost-effectiveness of targeted dengue vector interventions.^8^

## Methods

Upon development of a core protocol based on established multimethod approaches to public health research, and following an international call for letters-of-intent and a review of submissions by an external expert committee, multidisciplinary teams in six research institutions of South Asia (India, Sri Lanka) and South-East Asia (Indonesia, Myanmar, Philippines, Thailand) joined the initiative. The study design encouraged mutual complementarity among different research disciplines (i.e., interdisciplinarity) by combining eco-logical, bio-logical (i.e., entomological, epidemiological) and social (i.e., ‘eco-bio-social’) assessment of various dimensions affecting dengue in urban and peri-urban Asia.

All six sites first undertook a situation analysis to characterize and map the urban ecosystem, vector ecology in relation to rainfall, and social and cultural context, including stakeholder environment, community dynamics and gender implications. The situation analysis identified productive container types (i.e., those producing a large proportion of adult mosquito vectors), social and environmental risk factors favoring vector breeding, variation of vector ecology in the dry and wet season and in public and private spaces, and developed recommendations for locally adapted interventions. All six teams conducted a spatial analysis of randomly selected area clusters (urban neighborhoods determined by Geographic Information Systems grid sampling), with 20 clusters at three sites and 12 clusters at three other sites. The teams subsequently administered the following standardized research instruments that were based on methods in entomology, epidemiology, environmental sciences — including ecology — and social sciences:

1. household survey to assess basic demographic information (household composition), characteristics of housing and basic services, and knowledge of dengue and vector-related knowledge, attitudes and practices;
2. larval/pupal survey for use in households and public spaces, measuring water volume, water type, relative shading, location, usage, coverage, larval density, and pupae counts by container type;
3. cluster background information instrument to assess and describe the overall environmental situation of research clusters, including housing conditions and public spaces;
4. social research toolkit with modules that predominately utilize qualitative methods to assess: (1) social context and gender, (2) vector control policy and programme functioning, and (3) stakeholder environment;
5. standard guide for ecosystem characterization with reference to *Aedes* dengue vectors to facilitate the basic description of the ecosystem under study;
6. manual for applying Geographic Information Systems to dengue research to geo-reference clusters, households and open spaces.

The cross-site analysis^15^ published earlier facilitated deeper understanding of transmission dynamics. This formed the basis for teams at all sites to conduct a participatory problem analysis that included communities and other important stakeholder groups from public and private sectors and civil society in building consensus on potential intervention approaches. The articles in this special issue present insights revealed by detailed single-site data and analysis, while this overview article summarizes the overall research initiative and its findings across sites.

## Cross-site findings and development of site-specific intervention strategies

### Situation analysis

An important cross-site finding helped explain why rainfall is associated with increased dengue incidence by showing that uncovered outdoor water containers left unused for more than one week and usually positioned beneath vegetation were the most productive breeding sites of dengue vectors. Public spaces — except for schools and religious facilities — and commercial areas were much less important for pupal production than the peridomestic and intradomestic environment, particularly in densely populated neighborhoods. A complex yet non-significant association between water supply and pupal counts revealed that irregular supply with piped water, as well as the absence of piped water, may lead to increased water storage and vector infestation. Lack of waste disposal services was associated with vector abundance in only one site where, in the absence of large water containers, vectors bred in discarded containers (i.e., trash). Peoples’ knowledge of dengue transmitting mosquitoes was associated with reduced mosquito breeding and production, attributed to increased self-protection with domestic insecticides. Vector control measures (mainly larviciding in one site) substantially reduced the larval/pupal indices and ‘pushed’ mosquito breeding to alternative containers.

The comparative analysis of vector control services indicated that most vector control interventions are limited to space spraying and selective larviciding during local outbreaks and periods of increased case incidence, despite extensive dengue-related national or local guidelines and a significant formal organization of public surveillance and control services.

### Development of site-specific intervention strategies

All sites conducted a participatory problem analysis involving communities and other stakeholder groups, based on the situation analysis with important multisectoral stakeholder groups aiming at building consensus on potential intervention approaches. This process led to the design of site-specific intervention packages using innovative biological, chemical, mechanical and/or environmental vector control technologies and/or a combination of these tools. The intervention tools were selected according to the site-specific productive container types (Table 1) and ranged from mechanical lid covers for key productive water containers to bioproducts (e.g., Pyriproxyfen, Bti) to biological control agents (e.g., dragon fly nymphs, larvivorous fish, and copepods) (Tables 1–3). Several groups experimented with solid waste management, composting and recycling schemes particularly in those sites where small discarded water containers were the most productive ones.

**Table 1:** Productive containers for dengue vectors, type and delivery of interventions to reduce vector population

Country	Main productive containers	Type of intervention	Delivery through
India	Cement tanks, drums, barrels	Newly designed non-insecticidal water container covers	Womens groups
Indonesia	Bath water container, buckets, and small containers	Waste management Pyriproxyphen treatment	Community fora, volunteers, and city council
Myanmar	Cement tanks, drums, and small containers	Biological or mechanical control or pyriproxyphen	Partner groups and community
Philippines	Drums, barrels, and ceramic jars	Cleaning and larviciding of containers	Community volunteers and city council
Sri Lanka	Small discarded containers	Waste disposal	Waste collection services plus community
Thailand	Buckets, tyres, small discarded containers	Waste disposal, copepods, and Bti	Ecohealth volunteers

**Table 2:** Characteristics of study sites, community-based interventions and vector control approaches used (see Ref. 15 for a more detailed description of the study sites)

Research site	Urban ecosystem	Community interventions	Vector control approaches			
			Environmental, including community interventions	Mechanical tools	Biological control agents	Biological products
India (Chennai City)	Densely populated coastal city	Awareness raising among Women’s Self Help Groups	Source reduction, waste management and recycling	Elimination of key containers (e.g. grinding stones)		
		School-based IEC, in collaboration with ‘street health ambassadors’		Specially designed lids for cement tanks (wooden frames with polyester)		
		Student-initiated public awareness campaigns/rallies				
		IEC material distributed by ward volunteers				
Indonesia (City of Yogyakarta)	Rapidly urbanizing, densely populated city					
	Community forum			Zodia plantation (*Evodia suaveolens*), fish	Pyroproxyfen (applied city-wide by national NGO Tahija Foundation)	
		Culturally appropriate IEC material				
		School-based awareness programmes				
		Community empowerment, e.g., through establishment of environmental health forum (*Forum Lingkungan)*				
Myanmar (Yangon)	Two differently settled urban ecosystems (townships) of Myanmar’s capital city	Ward volunteers (*Yatkwet cetana-wundan*)	Waste management combined with distribution of IEC material (pamphlets and booklets) through *Thin-ga-ha* (friendship) groups for dengue vector control and environmental sanitation	Cotton Net Sweepers	Dragonfly Nymphs	Pyriproxyfen
		Distribution of existing IEC materials and the use of decision-making matrix tool for informed household-based selection of control tools		Lid covers		*Bacillus Thuringiensis Israelensis* (Bti)
Philippines (Masagana City)	Rapidly urbanizing city in Metro Manila	Barangay health care workers trained as outreach workers to monitor household management of water containers	Solid waste management	Drum lids (plain polyester nets)		
		IEC: educational DVD on dengue distributed in one cluster	Source reduction through household water container management			
			Health communication			
Sri Lanka (Gampaha district)	Semi-urban and rural district in close proximity to national capital city	Establishment of Environmental Health Association	Solid waste management, including improved waste collection using separation bags	Compost bins	Plants for home growing	
		Labor sharing (shramadarma)	Composters			
		Home gardening				
		Community mobilization				
		Community health volunteers				
Thailand (Chachoensao Province)	Urban and semi-urban areas of a provincial town and its outskirts	Establishment of ‘Ecohealth Club’ for community health volunteering	Source reduction and IEC efforts through EcoHealth volunteers (*Ar-sa-sa-makniwet sukkhaparb)*	Screen covers for earthen jars (*MosNet*)	Copepods	*Bacillus Thuringiensis Israelensis* (Bti)
				Mosquito trap (*MosHouse*)		
				Mosquito aspirator (*MosBuster*)		

**Note:** IEC, Information, Education, and Communication.

**Table 3:** Process indicators for community involvement (adapted from Ref. 16)

Indicator for community participation	Mobilisation	Collaboration	Empowerment
Leadership	Health professionals	Health professionals and community	Community

Planning and management	Health professionals tell community	Health professionals initiate, community participates	Professionals facilitate, community manages

Women’s involvement	Active participation not a programme objective	Women actively participate but have minor decision-making role	Active participation of women
External support	Funding from outside and controlled by health professionals	Majority of funding from outside but community contributes time, money and materials	Community finds ways to mobilising resources

Monitoring and evaluation	Health professionals	Joint M&E	Participatory community-driven M&E

### Empowerment of communities

**Figure 1:**
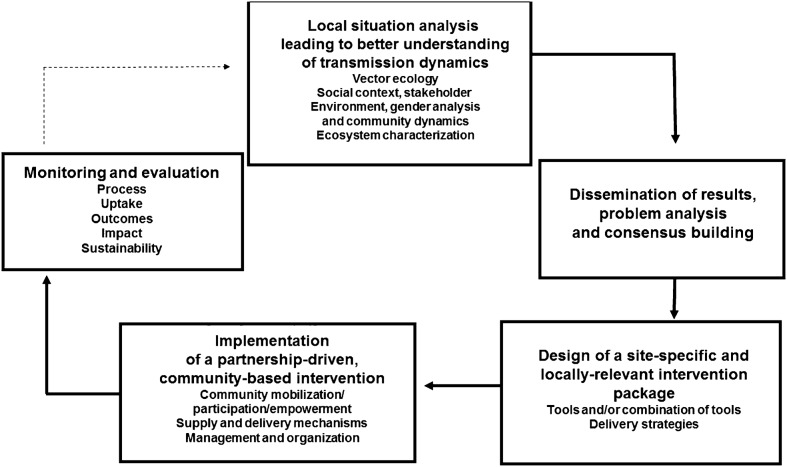
Process model for eco-bio-social research on integrated dengue vector control.

All sites developed socially and culturally appropriate health education materials. Various community groups (women’s groups, students, new volunteer groups for environmental health) were mobilized and empowered at different levels. The teams applied the process indicator framework for assessing degrees and intensities of community participation to their interventions^16^ (Table 3). The framework considers five key indicators for community participation, i.e., leadership, planning and management, women’s involvement, external support and monitoring and evaluation, and scored them on a 1–5 scale according to their degree of empowerment, collaboration and mobilization. Facilitated at a workshop, research teams self-assigned scores and mapped the respective intensity of community participation in spidergrams (Figure 1). All but one site (Philippines) reported very strong involvement of women in the intervention. Leadership by communities ranged widely from 1 to 5. The programmes led to significant outcomes at community level, with the formation of community groups and other public and private partners with broad environmental hygiene and sanitation interests.

### Intervention impact on vector densities

The impact on vector densities compared to control neighborhoods was significant in some sites (India, Thailand, Sri Lanka) and was as strong as in neighborhoods with enhanced public vector control but more sustainable in two sites (Myanmar, Indonesia).

## Discussion and conclusions

The common denominator in the six study sites was: (1) to design, conduct and evaluate multipartnership interventions with emphasis on community involvement; (2) to identify productive container types for adult dengue vectors (using pupal indices as a proxy measurement) and to apply a targeted approach in the productive containers; (3) to use as much as possible an ‘eco-health approach’ with judicious use or no use of insecticides according to IVM principles (integrated vector management)^17^ and (4) to assess — as far as the political and social conditions allowed — the effect of the intervention packages on partners and the vector populations.

**Figure 2:**
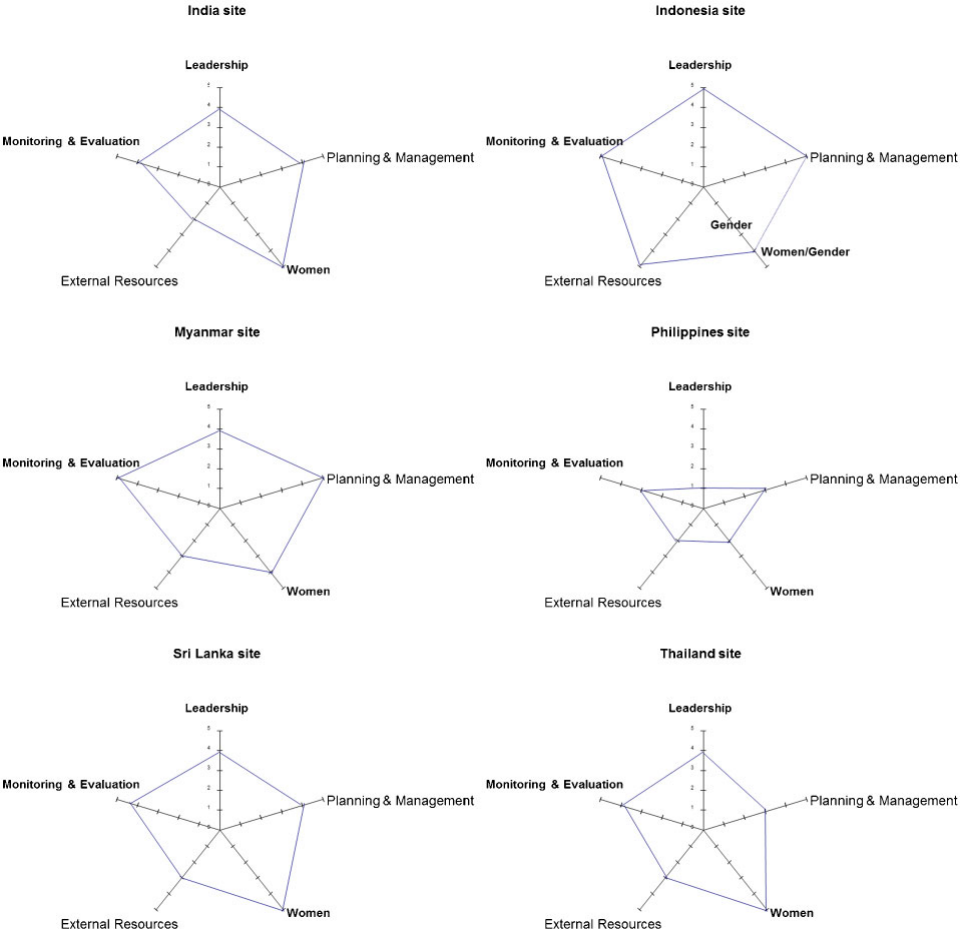
Spidergrams assessing five indicators of community participation for each research site.

Based on the cross-site process evaluation, a process model for eco-bio-social research on vector-borne diseases emerged and contains five basic elements necessary for locally relevant and community-based vector control (Figure 2):

- local situation analysis leading to better understanding of transmission dynamics through research on vector ecology, social context, stakeholder environment, gender analysis, community dynamics and ecosystem characterization;
- dissemination of early and formative results leading to collaborative problem analysis and consensus building;
- design of site-specific and locally-relevant intervention packages with combination of tools and clear delivery strategies;
- implementation of partnership-driven interventions through the mobilization, participation and empowerment of local communities and stakeholders at different levels, with clear definitions for supply and delivery mechanisms, as well as adequate management and organization;
- ongoing monitoring and evaluation of process, uptake, outcomes, impact and sustainability.

The prospects of a forthcoming dengue vaccine and — if ethically sound and acceptable by society — the use of genetically modified vectors will provide additional tools for achieving more comprehensive prevention of dengue virus transmission. Yet even with the future availability of such technical approaches, dengue prevention will continue to depend largely on innovative and robust intersectoral vector control strategies that are both cost-effective and sustainable. This study was unable to undertake a proper economic analysis of the cost-effectiveness and effect on dengue transmission dynamics of the intervention packages developed by the programmes in the initiative. However, evidence generated through this multicountry study suggests that vector management would be more sustainable when it complements or replaces other interventions by: (1) involving diverse partners — including local communities, (2) targeting water container interventions that achieve a significant reduction of dengue vectors (in India, Thailand, Sri Lanka and Myanmar), and (3) utilizing novel non-insecticidal intervention tools (such as rectangular water container covers in India, sweeping nets or dragon fly nymphs in Myanmar, and copepods and screen covers for earthen jars in Thailand). The findings are relevant for defining efficient, effective and ecologically sound vector control interventions that are based on local evidence and are in accordance with WHO’s strategy for IVM. Within this strategy, eco-bio-social research can be considered an important research framework for the systematic assessment of vector control needs and the development of partnership strategies at the local level.
